# Cost-per-responder analysis of patients with lenalidomide-refractory multiple myeloma receiving ciltacabtagene autoleucel in CARTITUDE-4

**DOI:** 10.3389/fimmu.2024.1408892

**Published:** 2024-08-21

**Authors:** Doris K. Hansen, Xiaoxiao Lu, Omar Castaneda Puglianini, Sonja Sorensen, Saad Z. Usmani, Eileen Zhang, Stephen Huo, Yan Zhang, Zaina P. Qureshi, Sundar Jagannath

**Affiliations:** ^1^ Department of Blood and Marrow Transplant and Cellular Immunotherapy, H. Lee Moffitt Cancer Center and Research Institute, Tampa, FL, United States; ^2^ Janssen Scientific Affairs, LLC, a Johnson & Johnson company, Horsham, PA, United States; ^3^ Evidera, Bethesda, MD, United States; ^4^ Memorial Sloan Kettering Cancer Center, New York, NY, United States; ^5^ Janssen Global Services, LLC, Raritan, NJ, United States; ^6^ Icahn School of Medicine at Mount Sinai, New York, NY, United States

**Keywords:** multiple myeloma, ciltacabtagene autoleucel, CAR T therapy, cost-per-responder analysis, cost-effectiveness analysis, daratumumab, pomalidomide, bortezomib

## Abstract

**Introduction:**

Ciltacabtagene autoleucel (cilta-cel) is a chimeric antigen receptor T-cell therapy approved for patients with relapsed/refractory multiple myeloma (RRMM). In the phase 3 trial, CARTITUDE-4 (NCT04181827), cilta-cel demonstrated improved efficacy vs. standard of care (SOC; daratumumab plus pomalidomide and dexamethasone [DPd] or pomalidomide plus bortezomib and dexamethasone [PVd]) with a ≥ complete response (≥CR) rate of 73.1% vs. 21.8%.

**Methods:**

A cost-per-responder model was developed to assess the value of cilta-cel and SOC (87% DPd and 13% PVd) based on the CARTITUDE-4 trial data from a US mixed payer perspective (76.7% commercial, 23.3% Medicare). The model was developed using progression-free survival (PFS), overall survival (OS), and ≥CR endpoints from CARTITUDE-4 over a period of 25.4 months. Inpatient stays, outpatient visits, drug acquisition, administration, and monitoring costs were included. The base-case model assumed an inpatient setting for each cilta-cel infusion; another scenario included 30% outpatient and 70% inpatient infusions. Costs of managing grade 3-4 adverse events (AEs) and grade 1-4 cytokine release syndrome and neurotoxicity were included. Subsequent therapy costs were incurred after disease progression; terminal care costs were considered upon death events. Outcomes included total cost per treated patient, total cost per complete responder, and cost per month in PFS between cilta-cel and SOC. Costs were adjusted to 2024 US dollars.

**Results:**

Total cost per treated patient, total cost per complete responder, and total cost per month in PFS were estimated at $704,641, $963,941, and $30,978 for cilta-cel, respectively, and $840,730, $3,856,559, and $42,520 for SOC over the 25.4-month period. Cost drivers included treatment acquisition costs before progression and subsequent treatment costs ($451,318 and $111,637 for cilta-cel; $529,795 and $265,167 for SOC). A scenario analysis in which 30% of patients received an outpatient infusion (assuming the same payer mix) showed a lower cost per complete responder for cilta-cel ($956,523) than those with an infusion in the inpatient setting exclusively.

**Discussion:**

This analysis estimated that cost per treated patient, cost per complete responder, and cost per month in PFS for cilta-cel were remarkably lower than for DPd or PVd, highlighting the substantial clinical and economic benefit of cilta-cel for patients with RRMM.

## Introduction

Multiple myeloma (MM) is the second most common hematologic malignancy and is characterized by abnormal monoclonal plasma cells in the bone marrow, extramedullary sites, or both (1, 2). In the United States (US), it is estimated that 35,780 new cases of MM will be diagnosed in 2024 and approximately 12,540 MM-related deaths will occur (3). Patients refractory to lenalidomide, often used continuously in front-line regimens, are unlikely to benefit from novel lenalidomide-based triplet chemotherapy regimens (4).

Ciltacabtagene autoleucel (cilta-cel) is a structurally differentiated B-cell maturation antigen (BCMA)-targeted chimeric antigen receptor (CAR) T-cell therapy (CAR T therapy) that received US Food and Drug Administration (FDA) approval in February 2022 for the treatment of patients with relapsed/refractory (RR) MM who have previously received four or more lines of therapy, including a proteasome inhibitor (PI), an immunomodulatory drug (IMiD), and an anti-CD38 monoclonal antibody, based on the results of the CARTITUDE-1 trial (NCT03548207) (5–7).

The follow-up phase III CARTITUDE-4 trial (NCT04181827) evaluated the efficacy and safety of cilta-cel vs. standard of care (SOC) therapy, consisting of either daratumumab, pomalidomide, and dexamethasone (DPd) or pomalidomide, bortezomib, and dexamethasone (PVd), in patients with MM refractory to lenalidomide who had received one to three prior lines of treatment, including a PI and an IMiD (8). Briefly, the CARTITUDE-4 trial was an open-label phase 3 randomized trial conducted at 81 sites in the US, Europe, Asia, and Australia. A total of 419 lenalidomide-refractory patients who had received between one and three lines of treatment for MM were randomized to receiving either a single cilta-cel infusion (n=208) or SOC (DPd [n=183, 87%] or PVd [n=28, 13%]) (8). Among patients assigned to receive cilta-cel, 10 (5.8%) never received cilta-cel as a trial treatment and 20 (9.6%) received cilta-cel after disease progression (8).

Progression-free survival (PFS) was significantly improved following cilta-cel infusion compared with trial SOC after a 16-month median follow-up (hazard ratio [HR], 0.26; P<0.01) (8). Cilta-cel also exhibited superior efficacy over SOC, with an overall response rate (ORR) of 84.6% vs. 67.3% and a complete response (CR) rate of 73.1% vs. 21.8%, respectively (8).

The process of administering cilta-cel involves many steps that are largely not required for SOC. First, patients’ blood is apheresed to collect mononuclear cells. Between the collection of patient cells and infusion of the CAR T product, bridging therapy may be administered if clinically indicated. Cilta-cel admistration is also preceded by lymphodepletion through a 3-day course of cyclophosphamide and fludarabine, as well as antipyretics and antihistamines administration 30 to 60 minutes prior to the infusion (5). Most CAR Ts are administered in the inpatient setting to monitor for the rapid onset of adverse events (AEs). However, cilta-cel’s generally predictable safety profile, including delayed onset of cytokine release syndrome (CRS) and immune effector cell-associated neurotoxicity syndrome (ICANS) [median CRS onset: 7-8 days (9) vs. 1-3 days for idecabtagene vicleucel [ide-cel] and axicabtagene ciloleucel (10–13); median ICANS onset: 8-9.5 days (5, 8) vs. 2-3 days for ide-cel and tisagenlecleucel (10)] facilitates its administration in the outpatient setting compared to other CAR Ts from a clinical and US insurance payer perspective (5, 14, 15). As a result, the administration of cilta-cel in the outpatient setting is expanding (16–19), as it is associated with several benefits including financial savings, expanded treatment access for patients, increased patient comfort, and better alignment with patient preferences who desire a prompt return to normalcy (15, 20, 21).

While the clinical efficacy of CAR T is remarkable, the high upfront cost of acquisition, alongside extensive procedures and facility costs, emphasizes the importance of conducting cost-effectiveness analyses (22). Therefore, this study assessed the value of cilta-cel compared to the CARTITUDE-4 SOC (DPd/PVd) using a cost-per-responder (CPR) model that incorporated efficacy and total treatment costs.

## Materials and methods

### Model overview and structure

A CPR model was developed in Microsoft Excel^®^ to compare the direct medical costs per patient receiving cilta-cel vs. SOC (DPd or PVd) using data from the CARTITUDE-4 trial, based on an intention-to-treat (ITT) approach. As such, the cost incurred for the entire patient journey is reported for all patients who entered the model (i.e., ITT population).

A 25.4-month time period was used, corresponding to the maximum observed follow-up for patients in the SOC arm (8, 23). The analysis was conducted from a mixed US payer perspective (76.7% commercial and 23.3% Medicare) (**Table 1**) (26).

**Table 1:** Key model inputs for the cost-per-responder analysis.

	Cilta-cel	SOC DPd/PVd
% of patients achieving CR or better	73.1%	21.8%
% of patients receiving CAR T infusion and lymphodepleting chemotherapy	84.6%*	–
% receiving bridging therapy (DPd or PVd)	100% (3 weeks)	–
Key AEs (23)		
CRS	63.5% (grade 1-2) 1.0% (grade ≥ 3)	0.5% (grade 1-2) 0% (grade ≥ 3)
CAR T-associated neurotoxicity (including ICANS)	14.9% (grade 1-2) 2.4% (grade ≥ 3)	0.0% (any grade)
Subsequent treatment distribution (23–25)		
CAR T		
Cilta-cel	0.0%	12.2%
Ide-cel	0.0%	17.9%
Triplet regimens		
DPd	15.2%	10.6%
DVd	9.2%	6.4%
DKd	11.8%	8.3%
IsaKd	11.6%	8.1%
EloPd	27.5%	19.2%
SVd	24.6%	17.2%

*Following an ITT approach, patients who received cilta-cel after disease progression (n=20, 9.6%) are additionally included in treatment costing.

AE, adverse event; CAR T, chimeric antigen receptor T-cell; Cilta-cel, Ciltacabtagene autoleucel; CR, complete response; CRS, cytokine release syndrome; DKd, daratumumab plus carfilzomib and dexamethasone; DPd, daratumumab plus pomalidomide and dexamethasone; DVd, pomalidomide plus bortezomib and dexamethasone; EloPd, elotuzumab plus pomalidomide and dexamethasone; ICANS, immune effector cell-associated neurotoxicity syndrome; Ide-cel, idecabtagene vicleucel; IsaKd, isatuximab, carfilzomib, and dexamethasone; PVd, pomalidomide plus bortezomib and dexamethasone; SOC, standard of care; SVd, selinexor, bortezomib, and dexamethasone.

Using PFS and overall survival (OS) curves from the CARTITUDE-4 trial (8, 23), the model partitioned the time in one of three states: PFS, post-progression survival (PPS), and death. Complete response rates were incorporated directly from the trial. The modeled outcomes included the total cost per treated patient, the total cost per complete responder, and the cost per month in PFS.

### Clinical inputs

Model clinical inputs, including PFS, OS, CR, and incidence of AEs were based on results from the CARTITUDE-4 trial (8, 23). PFS was used to determine the treatment duration for SOC, the proportion of patients progressing, and the time to start subsequent treatment for both arms.

Kaplan-Meier curves of the intention to treat population from CARTITUDE-4 were fitted to model the distributions of OS and PFS. PFS curves were fitted using a lognormal distribution for cilta-cel and SOC (**Supplementary Figure 1**); OS curves were fitted using a lognormal distribution for cilta-cel and a loglogistic distribution for SOC (**Supplementary Figure 2**).

Patients who progressed were assumed to receive a subsequent treatment. The list of potential subsequent treatment regimens, proportion of patients receiving each treatment regimen, and duration of subsequent regimens differed by treatment arm and was based on subsequent-line data from all three of the following sources: National Comprehensive Cancer Network guidelines (24) (to understand which regimens were recommended as subsequent treatment in US clinical practice), CARTITUDE-4 trial (23), and CancerMPact statistics (25) (the latter two sources were used to understand which regimens were used as a subsequent treatment and the proportion of patients receiving each subsequent regimen). The model assumed one line of subsequent treatment following progression on cilta-cel or SOC. Since the majority of deaths occurred after progression (and time to death was longer than time to progression based on **Supplementary Figures 1**, **2**), and since most pre-progression deaths were attributed to COVID-19, which is unrelated to MM and is unlikely to repeat in the future, the model assumed that all patients progressing received a subsequent treatment.

### Cost inputs

Patients in the cilta-cel arm accrued costs specific to the CAR T process, including apheresis, bridging therapy, pre-treatment lymphodepleting chemotherapy and post-infusion monitoring. For both treatment arms, drug acquisition, administration, co-medications (details are available in **Supplementary Tables 1A**, **1B**), monitoring (e.g., laboratory testing, vital sign assessments, and hematologist visits during PFS and PPS) and AE costs (including treatment-related costs and other resource use for AE management captured during the inpatient stay) were included. For the base-case model, patients received cilta-cel in the inpatient setting and incurred administration costs assuming a 7-day inpatient hospital stay followed by 7 outpatient days based on clinical assumptions and a double blinded Delphi panel of clinical experts (14). Post-infusion monitoring services during the first 112 days, accrued in addition of the initial administration services, were composed of bi-weekly hematologist visits, vital sign assessments three times per month, monthly laboratory testing, and one bone marrow biospy (14). After the first 112 days, monitoring services included monthly hematologist visits, vital sign assessments, and laboratory testing (14). Additional information on monitoring costs is available in **Supplementary Table 2A** and **Supplementary Table 2B**.

Costs of grade ≥3 AEs were accrued, except for CRS and CAR T-associated neurotoxicity (including ICANS), which were accrued for all grades, because these events are relevant to CAR T therapies and even lower grades of these events may have a considerable impact on resource use and associated costs. Cost for each AE was calculated based on the proportion of patients having the AE (based on CARTITUDE-4) (23) and the cost for a hospitalization related to this AE (14, 27, 28). Total AE-related costs were based on the sum of costs for each AE.

Post-progression costs included the cost of subsequent treatment, monthly monitoring (e.g., laboratory testing, vital sign assessments, and hematologist visits), and terminal care. The cost of subsequent treatments included the costs of treatment acquisition and administration for one additional line of therapy of median duration, and were incurred as a one-time cost at disease progression. Costs of terminal care were incurred upon death events. Post-progression costs per treated patient were calculated as the total post-progression costs based on the proportion of patients who progressed, divided by the total number of patients in the treatment arm using an ITT perspective).

The Medicare and commercial payer perspectives were evaluated using separated cost estimates. Centers for Medicare & Medicaid Services (CMS) Physician and Clinical Laboratory Fee Schedules, US commercial cost databases, and data collected from a targeted literature review were used to derive cost inputs (14, 27–37). All costs were reflective of 2024 or adjusted to 2024 US dollars based on the Consumer Price Index for medical care (38).

### Model outputs and scenario analyses

The total cost per treated patient (total cost of treatment divided by the ITT population) was reported for both cilta-cel and SOC arms, and included both pre-progression costs (i.e., apheresis [cilta-cel only], bridging therapy [cilta-cel only], pre-treatment lymphodepleting chemotherapy [cilta-cel only], infusion/administration, treatment acquisition, co-medications, monitoring post-infusion and during PFS, and adverse event costs) and post-progression costs (i.e., subsequent treatment, monitoring, and terminal care costs) (**Table 2**). Each cost component was calculated multiplying individual costs associated to a single process or event (i.e. total cost of one apheresis) by the proportion of patients incurring that specific cost. The total cost per complete responder (measured as the total cost per treated patient divided by the proportion of patients with complete response), and the cost per month of PFS (measured as the total cost per treated patient during PFS divided by the restricted mean number of months in PFS based on the fitted curves [i.e., 18.9 months for cilta-cel and 13.2 months for SOC]) were reported for both the cilta-cel and SOC arms.

**Table 2:** Base-case cost summary over 25.4 months.

Cost Category		Cilta-cel	SOC DPd/PVd	Source(s)
**Pre-progression costs**	Apheresis	$ 356	–	(30, 31)
	Bridging therapy	$ 46,496	–	(32, 33)
	Pre-treatment lymphodepleting chemotherapy	$ 3,379	–	(32, 33)
	Infusion/administration	$ 22,631	$ 5,218	(14, 30, 34, 36)
	Treatment acquisition	$ 451,318	$ 529,795	(32, 33)
	Co-medications	$ 10	$ 9	(32)
	Monitoring cost post-infusion	$ 6,266	-	(29–31)
	Monitoring during PFS	$ 9,552	$ 9,092	(14, 29–31)
	Adverse events	$ 44,180	$ 15,738	(14, 27, 28)
	**Total cost during PFS per treated patient**	**$ 584,189**	**$ 559,851**	
**Post-progression and other costs**	Subsequent treatment	$ 111,637	$ 265,167	(32, 33)
	Monitoring during PPS	$ 1,692	$ 5,346	(14, 29–31)
	Terminal care	$ 7,123	$ 10,366	(35, 37)
	**Total cost during PPS per treated patient**	**$ 120,451**	**$ 280,879**	
**Total cost per treated patient**		**$ 704,641**	**$ 840,730**	

Cilta-cel, Ciltacabtagene autoleucel; DPd, daratumumab plus pomalidomide and dexamethasone; PFS, progression-free survival; PPS, post-progression survival; PVd, pomalidomide plus bortezomib and dexamethasone; SOC, standard of care.

Scenario analyses were conducted to 1) explore an alternative payer perspective mix (i.e., 31% commercial and 69% Medicare) (39), which may be more representative of the population eligible to receive cilta-cel and 2) assess the impact of considering the outpatient infusion setting (70% inpatient setting and 30% outpatient setting and using the payer mix from the base-case analysis). For the latter scenario analysis, infusions in the outpatient setting accrued the cost of one inpatient day and 11 outpatient days (instead of 7 inpatient days and 7 outpatient days as used for the base-case model) (14).

## Results

### Base-case analysis

Over the course of the 25.4-month time period, the total cost per treated patient with cilta-cel was estimated to be $704,641, while total cost of SOC was estimated to be $840,730 (**Table 2**). For both arms, treatment acquisition costs represented most of the total cost per treated patient. Prior to progression, the cost of cilta-cel per treated patient was higher than cost of SOC ($584,189 vs. $559,851), which was primarily driven by higher costs associated with the infusion procedure (e.g., cilta-cel administration, post-infusion monitoring) and costs of AEs. Following progression, costs of cilta-cel per treated patient were lower ($120,451 vs. $280,879), which was primarily driven by lower subsequent treatment costs ($111,637 vs. $265,167).

**Figure 1:**
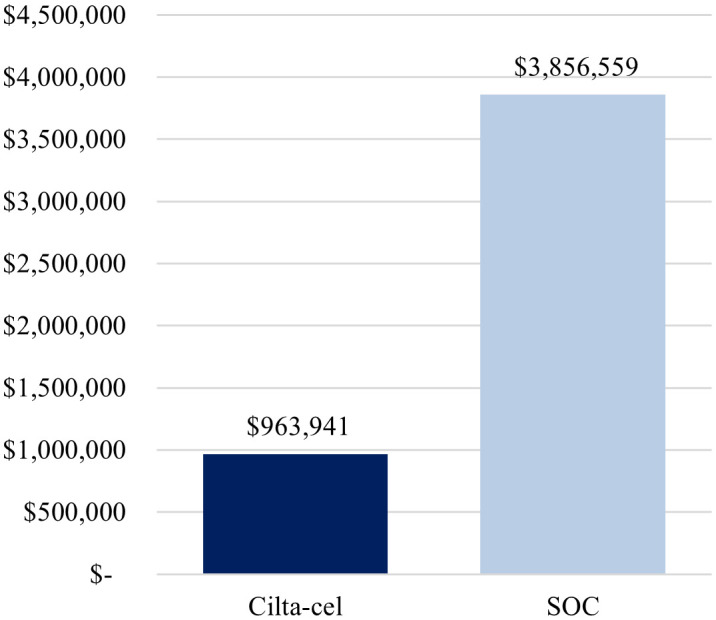
Base-case cost per complete responder. Cilta-cel, Ciltacabtagene autoleucel; SOC, standard of care.

**Figure 2:**
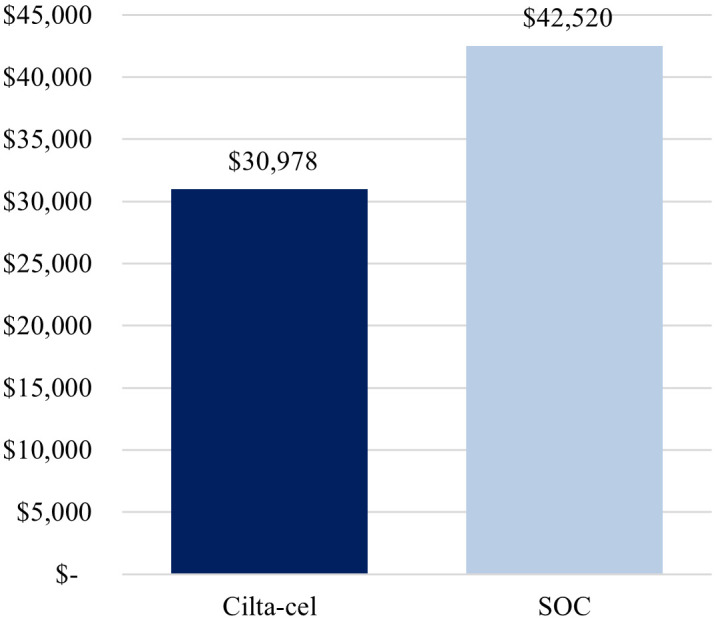
Base-case cost per month in PFS. Cilta-cel, Ciltacabtagene autoleucel; PFS, progression-free survival; SOC, standard of care.

Total cost per complete responder was lower for cilta-cel compared with SOC ($963,941 vs. $3,856,559) (**Figure 1**), driven by the higher complete response rate observed for cilta-cel vs. SOC (73.1% vs. 21.8%). Similarly, the cost per month in PFS was estimated to be lower for cilta-cel compared to SOC ($30,978 vs. $42,520) (**Figure 2**).

### Scenario analyses

**Figure 3:**
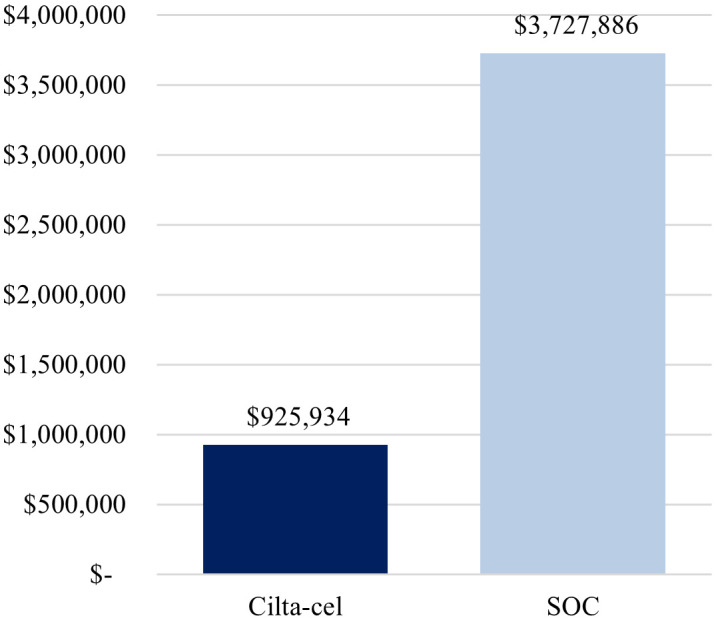
Cost per complete responder (alternative payer mix: 69% Medicare, 31% commercial). Cilta-cel, Ciltacabtagene autoleucel; PFS, progression-free survival; SOC, standard of care.

**Figure 4:**
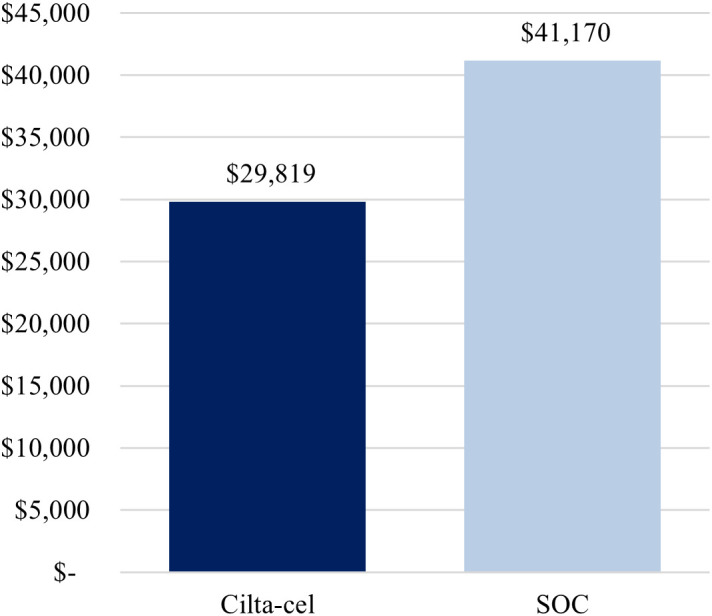
Cost per month in PFS (alternative payer mix: 69% Medicare, 31% commercial). Cilta-cel, Ciltacabtagene autoleucel; PFS, progression-free survival; SOC, standard of care.

The first scenario analysis, assuming a 69%-Medicare and 31%-commercial payer mix, yielded similar conclusions as the base-case analysis, whereby treatment with cilta-cel yielded a lower cost per complete responder compared to SOC ($925,934 vs. $3,727,886) (**Figure 3**), as well as a lower cost per month in PFS ($29,819 vs. $41,170) (**Figure 4**).

The second scenario analysis, assuming that 30% of the cilta-cel cohort received their infusion in the outpatient setting (and that the payer mix is the same as in the base-case analysis), yielded a lower administration cost ($17,209 vs. $22,631 for base-case model), resulting in a lower total cost per treated cilta-cel patient ($699,218 vs. $704,641 for base-case model) and a lower cost per complete responder compared to SOC ($956,523 vs. $963,941 for base-case model).

## Discussion

The CPR analysis of data from the CARTITUDE-4 trial indicated that, among patients with lenalidomide-refractory MM, the cost per complete responder and cost per month in PFS with cilta-cel treatment were remarkably lower than those of SOC therapy (DPd/PVd) over 25.4 months. Additionally, cilta-cel treatment resulted in lower total costs per treated patient compared to SOC over the same period.

Cilta-cel treatment resulted in greater total costs pre-progression ($584,189) compared to SOC ($559,851), reflecting the additional resources associated with CAR T-cell therapy administration, including apheresis, bridging therapy, and post-infusion monitoring that are not required for SOC. However, total post-progression costs were substantially lower with cilta-cel treatment ($120,451) compared to SOC ($280,879) during the time period analyzed, due to the greater efficacy of CAR T and associated reduced need for subsequent treatments (8, 40). Indeed, the differentiating attribute of cilta-cel is its impressive response rate relative to other treatments available, which delays progression and helps patients avoid cycling through multiple lines of subsequent therapy (7, 8, 41).

The CAR T-cell infusion setting also impacts cost-effectiveness of treatment, largely due to the high hospitalization costs associated with inpatient monitoring for common post-infusion AEs including CRS and ICANS (16). The improved safety profile of novel CAR T-cell therapies, delayed onset of CRS and ICANS relative to earlier CAR T-cell treatments, and enhanced ability to manage common post-infusion AEs have all increased the interest and feasibility of outpatient CAR T-cell infusion (40). In the current study, a scenario analysis in which 30% of patients received CAR T-cell infusion in the outpatient setting (assuming an outpatient setting of care immediately following the infusion and the same payer mix as the base-case analysis) resulted in a $7,418 reduction in the cost per complete responder relative to the base-case analysis. Similarly, post-treatment costs based on the CARTITUDE-1 trial were estimated over a 12-month period for outpatient cilta-cel administration, with subsequent cost reductions of $2,838 and $5,677 per patient when 15% or 30% of patients received outpatient infusion, respectively (14). This reduced economic burden can be achieved while maintaining safety and efficacy outcomes, with a recent systematic review identifying similar safety and efficacy rates following CAR T-cell infusion in either the inpatient or outpatient setting for patients with MM, lymphoma, or acute lymphoblastic leukemia (16). To note, our study assumed that “outpatient administration” of cilta-cel consisted of one day in the inpatient setting followed by 11 days in the outpatient setting (as opposed to seven days inpatient followed by seven outpatient days for the base-case model). However, the exact distribution of days in the inpatient and outpatient settings associated with an outpatient administration is expected to differ based on several patient-, physician- and facility-specific factors (42). Therefore, the magnitude of the economic benefit of outpatient administration of cilta-cel may also differ accordingly.

While, to the best of our knowledge, no other CPR analysis has been conducted for cilta-cel, an evaluation of CAR T-cell therapy ide-cel, the only other CAR T approved for patients with relapsed or refractory MM, estimated a cost of $1,710,000 per complete responder and $50,000 per month in PFS (43). This may be partially explained by a greater proportion of patients achieving CR or better when on cilta-cel as observed in clinical trials (73.1% at median follow-up time of 15.9 months) (8) than on ide-cel (39% at median follow-up time of 18.6 months) (44). This hypothesis is also supported by a recent matching-adjusted indirect comparison of cilta-cel vs. ide-cel, which found that patients in the cilta-cel group were significantly more likely to achieve complete response or better (response ratio: 1.91 [95% CI: 1.56, 2.34]) and less likely to progress or die than patients in the ide-cel group (HR: 0.51 [95% CI: 0.31, 0.84]) (45).

This study was subject to some limitations, most of which are inherent to cost modeling studies. First, inputs and assumptions used in the study model were based on published literature and therefore may be subject to some level of uncertainty or may not be applicable to all situations. For example, patients receiving cilta-cel were assumed to be consistently seen bi-weekly during the first 112 days and monthly thereafter based on published data (14), but variations of this frequency may occur. In addition, for most AEs (including cytopenias), AE-related costs were based on inpatient costs for these AEs (including costs specific to grade 1-2 and grade ≥3 CRS/ICANS), but some costs for services and treatments received outside of the inpatient setting (e.g., for patients remaining cytopenic for several months and requiring transfusion support) may not be captured. Second, clinical inputs, including PFS, OS, CR, and AE rates, were based on results from the CARTITUDE-4 trial and are not necessarily fully reflective of real-world outcomes, potentially limiting generalizability of the study findings. Third, as the time period considered was 25.4 months (the maximum observed follow-up for patients in the SOC arm), we had limited visibility into the cost-effectiveness of cilta-cel vs. SOC over a longer time horizon. However, since the cost-effectiveness of CAR T seems to increase over time due to its prolonged PFS (46), we would expect the difference in costs between arms would likewise increase. Finally, the model assumed that one line of subsequent treatment was considered for each patient post progression. However, it is possible that some patients did not receive any subsequent treatment after progression, and that others received more than two subsequent regimens during the 25.4-month follow-up period. Therefore, the impact of this assumption on our findings is unclear.

Using a CPR analysis, this study demonstrated the cost-effectiveness of cilta-cel compared to CARTITUDE-4 trial’s SOC, identifying remarkably lower costs per treated patient, costs per complete responder, and costs per month in PFS for patients treated with cilta-cel. These findings highlight the importance of considering treatment effectiveness, as well as long-term cost, when evaluating treatment options for patients with RRMM.

## Data Availability

The original contributions presented in the study are included in the article, further inquiries can be directed to the corresponding author/s. Requests to access the datasets should be directed to zquresh3@its.jnj.com.
