# Supplementary material for: Cost-per-responder analysis of patients with lenalidomide-refractory multiple myeloma receiving ciltacabtagene autoleucel in CARTITUDE-4
Source: Front Immunol. 2024 Aug 21;15:1408892. doi: 10.3389/fimmu.2024.1408892 (PMC11372240; doi:10.3389/fimmu.2024.1408892)
Supplement: Supplementary file 1 [file DataSheet1.docx]

Supplementary Material

Cost per Responder Analysis of Patients with Lenalidomide-Refractory Multiple Myeloma Receiving Ciltacabtagene Autoleucel in CARTITUDE-4

Doris K. Hansen^*^, Xiaoxiao Lu, Omar Castaneda Puglianini, Sonja Sorensen, Saad Z. Usmani, Eileen Zhang, Stephen Huo, Yan Zhang, Zaina P. Qureshi, Sundar Jagannath

***Correspondence:** Doris K Hansen, MD. [Doris.Hansen@moffitt.org](mailto:Doris.Hansen@moffitt.org)

**Supplementary Figure 1. Fitted PFS curves**

**Abbreviations:** Cilta-cel = Ciltacabtagene autoleucel; PFS = progression-free survival; SOC = standard of care.

**Supplementary Figure 2. Fitted OS curves**

**Abbreviations:** Cilta-cel = Ciltacabtagene autoleucel; OS = overall survival; SOC = standard of care.

**Supplementary Table 1a. Co-medications: CAR T-related prophylactic and other medications**

| **CAR-T Related prophylactic and other medications** | **Total cost per course of prophylaxis** | **Notes** |
| --- | --- | --- |
| Antiviral - Acyclovir | $8.85 | 800 mg 5 times daily for 7 days  (based on US prescribing information for herpes zoster) |
| Antibacterial - Sulfamethoxazole; trimethoprim | $1.26 | 1 tablet (DS tablet) of 800 milligrams (mg) of sulfamethoxazole and 160 mg of trimethoprim every 12 hours for 10 days |
| Antihistamine - Diphenhydramine | $0.02 | One time pre-infusion |
| Antipyretic - Acetaminophen | $0.02 | One time pre-infusion |
| **Total cost of co-medication** | **$10.14** |  |

**Abbreviations:** CAR T: chimeric antigen receptor T-cell therapy.

**Sources:** CARTITUDE-4 CSR, US prescribing information, Merative. Micromedex® RED BOOK®.

**Supplementary Table 1b. Co-medications: SOC-related prophylactic and other medications**

| **Co-medication** | **Pack size** | **Strength** | **Price per pack** | **Dosage per administration** | **Total cost per administration** |
| --- | --- | --- | --- | --- | --- |
| Antiviral - Acyclovir | 100 | 400 mg | $12.64 | 800 mg | $0.25 |
| Antibacterial - Sulfamethoxazole; trimethoprim | 100 | 800 mg | $6.30 | 800 mg | $0.06 |
| Antihistamine - Diphenhydramine | 100 | 50 mg | $1.75 | 50 mg | $0.02 |
| Antipyretic - Acetaminophen | 100 | 325 mg | $0.77 | 650 mg | $0.02 |
| **Total cost of co-medication for each daratumumab/pomalidomide administration** |  |  |  |  | **$0.35** |
| **Total cost of co-medication for SOC over entire course** |  |  |  |  | **$8.75** |

**Abbreviations:** SOC = standard of care.

**Sources:** CARTITUDE-4 CSR, US prescribing information, Merative. Micromedex® RED BOOK®

**Supplementary Table 2a. Monitoring cost post-infusion – Cilta-cel**

| **Hospital and laboratory monitoring** | **Unit cost** | **Weekly resource use post-infusion (first 112 days)** | **Weekly resource use after 112 days pre-progression** | **Weekly resource use post progression** |
| --- | --- | --- | --- | --- |
| **Hematologist visit** | $140.48 | 1.96 | 0.25 | 0.25 |
| **Vital signs, including oxygen saturation** | $12.12 | 0.70 | 0.25 | 0.25 |
| **Laboratory testing** |  |  |  |  |
| Complete blood count | $30.96 | 0.70 | 0.25 | 0.25 |
| Uric acid assay | $35.72 | 0.70 | 0.25 | 0.25 |
| Serum free light chains and serum/urine immunofixation | $147.87 | 0.21 | 0.25 | 0.25 |
| Quantitative immunoglobulin | $48.95 | 0.21 | 0.25 | 0.25 |
| Serum M-protein quantitation by electrophoresis | $66.16 | 0.21 | 0.25 | 0.25 |
| 24-hour urine protein electrophoresis sample | $93.89 | 0.21 | 0.25 | 0.25 |
| Serum calcium corrected for albumin | $19.61 | 0.70 | 0.25 | 0.25 |
| Bone marrow biopsy | $370.88 | 0.07 | 0.00 | 0.00 |
| **Total monitoring weekly cost** |  | **$445.13** | **$148.94** | **$148.94** |
| **Total monitoring post-infusion** |  | **$6,265.67^1^** | **$9,552.37^2^** | **$1,691.90^3^** |

^1^ Calculated for the pre-progression period, for the first 112 days.

^2^ Calculated for the pre-progression period, after the first 112 days.

^3^ Calculated for the post-progression period.

**Abbreviations:** Cilta-cel = Ciltacabtagene autoleucel.

**Sources:**

Jagannath S, Joseph N, Crivera C, et al. Component Costs of CAR-T Therapy in Addition to Treatment Acquisition Costs in Patients with Multiple Myeloma. *Oncol Ther*. Jun 2023;11(2):263-275. doi:10.1007/s40487-023-00228-5

Centers for Medicare & Medicaid Services (CMS.gov). Clinical Diagnostic Laboratory Fee Schedule <https://www.cms.gov/medicare/payment/fee-schedules/clinical-laboratory-fee-schedule-clfs/files>

Centers for Medicare and Medicaid Services (CMS.gov). Physician fee schedule look-up tool. <https://www.cms.gov/medicare/physician-fee-schedule/search>

Practice Management Information Corporation (PMIC). Medical Fees Directory 2024. Usual, customary and reasonable (UCR) fees. ISBN: 978-1-57066-439-7.

**Supplementary Table 2b. Monitoring cost – SOC**

| **Hospital and laboratory monitoring** | **Unit cost** | **Weekly resource use pre-progression** | **Weekly resource use post-progression** |
| --- | --- | --- | --- |
| **Hematologist visit** | $140.48 | 0.25 | 0.25 |
| **Vital signs, including oxygen saturation** | $12.12 | 0.25 | 0.25 |
| **Laboratory testing** |  |  |  |
| Complete blood count | $30.96 | 0.25 | 0.25 |
| Uric acid assay | $35.72 | 0.25 | 0.25 |
| Serum free light chains and serum/urine immunofixation | $147.87 | 0.25 | 0.25 |
| Quantitative immunoglobulin | $48.95 | 0.25 | 0.25 |
| Serum M-protein quantitation by electrophoresis | $66.16 | 0.25 | 0.25 |
| 24-hour urine protein electrophoresis sample | $93.89 | 0.25 | 0.25 |
| Serum calcium corrected for albumin | $19.61 | 0.25 | 0.25 |
| Bone marrow biopsy | $370.88 | 0.02 | 0.02 |
| **Total monitoring weekly cost** |  | **$156.07** | **$156.07** |
| **Total monitoring post infusion** |  | **$9,091.56^1^** | **$5,345.65^2^** |

^1^ Calculated for the pre-progression period.

^2^ Calculated for the post-progression period.

**Abbreviations:** SOC = standard of care.

**Sources:**

Jagannath S, Joseph N, Crivera C, et al. Component Costs of CAR-T Therapy in Addition to Treatment Acquisition Costs in Patients with Multiple Myeloma. *Oncol Ther*. Jun 2023;11(2):263-275. doi:10.1007/s40487-023-00228-5

Centers for Medicare & Medicaid Services (CMS.gov). Clinical Diagnostic Laboratory Fee Schedule <https://www.cms.gov/medicare/payment/fee-schedules/clinical-laboratory-fee-schedule-clfs/files>

Centers for Medicare and Medicaid Services (CMS.gov). Physician fee schedule look-up tool. <https://www.cms.gov/medicare/physician-fee-schedule/search>

Practice Management Information Corporation (PMIC). Medical Fees Directory 2024. Usual, customary and reasonable (UCR) fees. ISBN: 978-1-57066-439-7.
